# Nudging Altruism by Color: Blue or Red?

**DOI:** 10.3389/fpsyg.2019.03086

**Published:** 2020-01-22

**Authors:** Xinyu Nie, Han Lin, Juan Tu, Jiahe Fan, Pingping Wu

**Affiliations:** ^1^Antai College of Economics and Management, Shanghai Jiao Tong University, Shanghai, China; ^2^School of Information Engineering, Jiangsu Key Laboratory of Auditing Information Engineering, Nanjing Audit University, Nanjing, China; ^3^School of Physics, Nanjing University, Nanjing, China

**Keywords:** altruism, color, experiment, behavior, decision heuristic

## Abstract

Altruism can be spontaneously aroused by environmental factors. However, the mechanism behind these factors is subject to debate. We carried out a study of laboratory experiment using computer-based Mouselab method to determine the mechanism. We found that different colors altered the altruistic behaviors of people. Specifically, blue enhanced altruism, whereas red discouraged altruism. We used a process-tracing technique to monitor the selection of an adaptive strategy and demonstrate that different colors can simulate changes in information acquisition and then lead to the corresponding behaviors. The results suggested that the decision heuristic plays a mediating role in the link between colors and individual altruistic behaviors.

## Introduction

Altruism is a central issue in our evolutionary origins, social relations, and societal organization (Fehr and Fischbacher, 2003). Previous studies divided altruism into three main categories: people in the first category, who help others at their own expense, may be in a position of empathy or embody other positive emotions (e.g., Batson, 1991; Branas-Garza, 2007). The second category, referred to as altruistic punishment, is usually used to maintain social fairness and cooperation (e.g., Boyd et al., 2003). People who violate norms are punished at a high cost. The third category of altruism is closely related to morality, and the benefits of such moral altruistic behavior tend to favor stronger moral values (e.g., Capraro and Vanzo, 2019; Capraro et al., 2019).

Among the numerous altruistic behaviors, donation has always been a part of our daily lives from donating toys as children to making anonymous donations to charities as adults. The donations of Americans to charities totaled $427.71 billion in 2018, according to the Giving USA Foundation. Moreover, studies on altruistic and antisocial behavior have shown that without exception, altruistic behavior is influenced by the environment (Krueger et al., 2001). Thus, nudging or promoting altruism (e.g., donation behavior) through environmental factors is cheaper and more effective than using punishment or rewards to promote altruism.

Color, one of the environmental factors that influences altruism, appears everywhere from web page backgrounds to product packaging. Different colors fill our daily lives, but we never attribute behavioral shifts to them. Related color studies have concluded that wavelength is the major cause of color effects (e.g., Stone and English, 1998). For example, blue light has been found to activate the melanopsin photoreceptor system to stimulate brain structures involved in sub-cortical arousal and higher order attentional processing (Cajochen et al., 2005).

Red and blue have been compared most often in previous studies because of their opposite positions in the color spectrum. For example, a study of animal behavior suggested that red signals vigor and dominance and induces aggression (Cuthill et al., 1997; Andersson et al., 2002). Studies on the effects of color on physiological data have also shown that red is more likely to cause arousal than green (Wilson, 1966), and blue calms people more than red (Jacobs and Hustmyer, 1974; Gorn et al., 2004). In addition, in a recent study, the willingness to pay in auctions and negotiations demonstrated the effects of red and blue, with the two mediators being arousal and aggression (Bagchi and Cheema, 2013). Thus, we proposed that red and blue also affect donation behavior. Specifically, we proposed that blue makes people more altruistic than blue.

The mechanisms behind the effects on altruism vary. Individual decision-making strategies affect most human behavior. Given the different processing strategies, information acquisition plays a key role in continually affecting human behavior. Considering the multiple dimensions of color effects, the influence of color on behavior may also be driven by decision-making strategies, such as information acquisition. In previous research, red enhanced attention during detail-oriented tasks (Mehta and Zhu, 2009), whereas blue set the tone for creative tasks (Hatta et al., 2002). These findings revealed different patterns of information acquisition. Thus, our study attempted to explore whether information acquisition is a mediating variable between colors and altruism.

Consumers can acquire information in an attribute- or alternative-based manner (Payne et al., 1988; Jang and Yoon, 2016). A study of the consumer choice demonstrated that the attribute-based pattern resulted in behavioral shifts driven by desirability-related factors (e.g., design), whereas the alternative-based pattern resulted in behavioral shifts driven by feasibility-related factors (e.g., price; Pizzi et al., 2014). The shifts could be ascribed to different construal levels (Lynch and Zauberman, 2007). According to the Construal Level Theory (CLT), individuals adopt a low-level construal (i.e., a concrete way to explain psychologically proximal events) and a high-level construal (i.e., an abstract way to explain psychologically distal events; Borovoi et al., 2010). For example, consider students completing assignments in the classroom. When it is a psychologically proximal event, a lower level construal may contain more details on the activity, such as students’ ages and genders, indoor temperature, and the specific content of the assignment. When it is a psychologically distal event for the same activity, a higher level construal may be “work hard.” There has been some proof of this phenomenon in recent research. For example, CLT has been found to influence economic behaviors to a great degree in areas such as retirement savings and bank account openings (Leiser et al., 2008). Our purpose was in accordance with the stream of research that CLT can explain different patterns of information acquisition that influence human behavior.

Product attributes are general and abstract features that vary individually, and products are gradually difficult to extract from the context. As such, information on the same object-based on attributes and alternatives may yield different construal levels (Borovoi et al., 2010). Attribute-based patterns represent abstract and high-level construals, whereas alternative-based patterns represent concrete and low-level construals (Pizzi et al., 2014; Lee et al., 2017). Different construal levels produce different consumer purchasing behaviors, even within the same choice set (Lynch and Zauberman, 2007). In a previous study of episodic future thinking, altruism was enhanced at high construal levels (Yi et al., 2016). The current research proved the effects of color on altruism, with the mediating variable being information acquisition.

Through two laboratory experiments, we demonstrated that color nudged altruism, with the mediating variable being information acquisition. The whole experiment was conducted on a computer. First, the background color was changed to red or blue. Then, we followed a method acquired from a study on decision-making (Payne et al., 1988), the Gamble test, to measure the behavioral pattern that reflected the information acquisition (a more alternative-based or attribute-based manner). Finally, a picture and a scenario of a charity theme followed by a question about donation were adopted to measure the altruism. The results of our study showed that blue enhanced altruistic behavior more significantly compared to red. Furthermore, red induced a more alternative-based pattern relative to blue, which caused individuals to donate less.

## Method

We conducted the experiment in a laboratory, and all participants were recruited from Nanjing University and Shanghai Jiao Tong University in China. In addition, our study was approved by the human research ethics committee for non-clinical faculties at Nanjing University before the experiment began. Computer-based experiments were conducted to investigate the influence of colors on altruism and the underlying mechanism. All participants were assigned to a consistent computer to ensure that the effects of computer display differences were eliminated.

First, the entire process was performed with a random background color (red or blue) for each participant. The hue-saturation-lightness (HSL) scheme, which was introduced by Mehta and Zhu (2009), was used in the experiments (red: hue = 0, saturation = 240, lightness = 120; blue: hue = 160, saturation = 240, and lightness = 120). Throughout the experiment, red or blue background colors filled the screen. Before the experiment began, we issued a statement that this was a study using an anonymous form to focus on human behavioral shifts under different colored backgrounds. The data were only used for scientific research. All volunteers were shown the ethical approval and independently made the decision about whether to participate in the experiment. Then, all participants were informed about consent and signed a written consent form. Moreover, all the participants were told that no personal information would be recorded and there would be no judgment of objectively “right” or “wrong.” as each choice completely relied on the individual’s subjective feeling.

**FIGURE 1:**
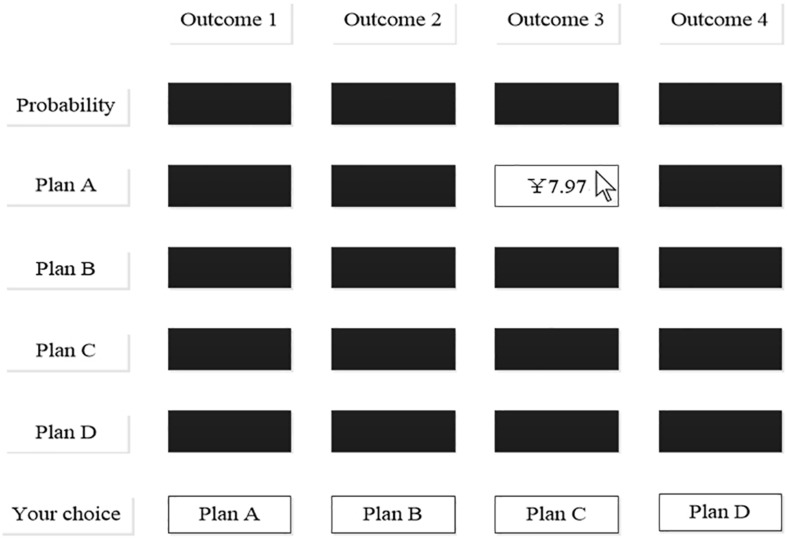
Gamble game using a process-tracing technique.

Second, the Gamble test (Payne et al., 1988) was used to test individual decision heuristic patterns. This game reflected the pattern of information acquisition by comparing the attributes of the same alternative or the alternatives of the same attribute. In the Gamble game, the matrix of available information was presented on a computer screen. The probabilities of the four outcomes appeared in the first row, and the payoffs correlated with each outcome appeared in the second row. Then, the next four rows contained the information about the payoffs associated with the different outcomes for each alternative. Finally, four alternatives at the bottom were the options for subjects to choose. During the decision-making process, all the areas outside the boxes were filled with blue or red (see Supplementary Appendix). Four risky options were included in one set, and each option had a possible payoff ranging from ¥0.01 to ¥9.99. All the options had the same four outcome probabilities, ranging from 0.01 to 0.96 (corresponding to the four black boxes in the first row in Figure 1), whereas the probabilities of each option equaled one. In terms of the design used for the simulation study, sets of options were sampled from the random-dispersion, dominance-possible conditions. For example, one set of gambles had probabilities of 0.38, 0.25, 0.16, and 0.21 for the four possible outcomes. Plan A provided payoffs of ¥8.76, ¥3.73, ¥7.97, and ¥2.21, respectively, for the four outcomes (corresponding to the four black boxes in the second row in Figure 1). Plan B provided payoffs of ¥5.21, ¥6.32, ¥6.83, and ¥8.32, respectively (corresponding to the third row in Figure 1). Plan C and D had different but similar types of payoffs (corresponding to the fourth and fifth row in Figure 1). Thus, the average expected values of the options in the random-dispersion conditions were equivalent to those under different conditions.

Through this form, five sets of options were presented. The subjects could acquire information about probabilities and payoffs and then make a decision (e.g., click “plan A” on the bottom row). The complete experimental session took 5–15 min. When the first set appeared, all the values were “hidden” in the labeled boxes (corresponding to the black boxes in Figure 1). When the cursor moved into the box, the corresponding value appeared until the cursor was moved out (corresponding to the boxes with “¥7.97” in Figure 1). It’s worth noting that you could only read one box at a time. In addition, data from the five sets, including time spent, were recorded.

After the gamble test, the participants were shown a picture of an underprivileged learning (see Supplementary Appendix) and told to read a scenario that described how many of such children lack basic educational resources. The scenario noted that a non-profit, philanthropic organization that provided free educational resources or services, were asking people to donate money to buy educational materials or to provide free tutoring and other educational services for the children in need. After being shown the information, participants were asked to report their willingness to donate money to the charity as follows: “How much would you be willing to donate if you recently received an extra ¥100 income? (It can be zero.)” and “How much discretionary money do you have every month?” The amount that the subjects were willing to donate and the discretionary money per month were recorded.

In this section, we only provided a single response choice: a charitable organization that provides educational resources for poor children. A study on donations showed that people were more willing to donate to a charity than to a particular victim (Ein-Gar and Levontin, 2013). Thus, our study selected one charitable organization as the object of donation. This study followed the theme used in most previous research on donation behavior: helping poor children. In addition, previous literature on donation behavior only used one donation target (Ongley et al., 2014; Bjälkebring et al., 2016), as too many targets could distract the participants. If there was a discrepancy between the participants and the charity’s goals, we determined that the difference should be the same under both conditions (red and blue).

A dummy variable was used to code a background color (0 = red and 1 = blue) as the independent variable. Gender, age, and living expenses were recorded as covariates. Monetary donations were the dependent variable, which expressed the altruistic behavior of the participants. The decision heuristic pattern as a mediated variable was calculated through decision processing. Information acquisition and decision behavior could be characterized in many ways. One could examine the amount and sequence of the information acquired, and the time spent acquiring information (Klayman, 1983). We followed Payne et al.’s method to calculate the behavior pattern, which ranges from a value of −1.0 to +1.0 (Payne et al., 1988):

(1) $$\text{PATTERN}=\frac{N_{a\,l\,t\,e\,r}-N_{a\,t\,t\,r}}{N_{a\,l\,t\,e\,r}+N_{a\,t\,t\,r}}$$

where *N*_*alter*_. denotes the amount of alternative-based transitions, and *N*_*attr*_ denotes the amount of attribute-based transitions. Both values related to the sequence of information acquisition. When you got a piece of information from an item (the number behind the “hidden” box) by clicking on it, it followed two cases of information acquisition. The first was the item that referred to the same alternative but a different attribute, defined as the alternative-based transition; the second item referred to a different alternative but the same attribute, defined as the attribute-based transition. *t*-Value conveyed a more alternative-based process when positive and a more attribute-based process when negative.

In addition, another similar replicating experiment (*n* = 88) was conducted as a robustness test to determine whether the effect could be generalized to time donations (as opposed to monetary donations). In the replicating experiment, the only difference was that participants reported their willingness to donate their time to the charity after reading about the charity project as follows: “How much time would you be willing to donate if you were to get two extra days off? (It can be zero.)” and “Please rate your schedule tightness level (1 = extremely loose, 7 = extremely tight).”

## Results

A total of 173 subjects participated in our study (85 in the monetary donation experiment and 88 in the time donation experiment). Table 1 presents the means, standard deviations, and correlations of all the variables in our experiment on monetary donation. In the monetary donation experiment, the age of the participants ranged from 18 to 45 (mean = 25.06, *SD* = 3.91), and 46% (*n* = 38) of them were female. The average time to complete the experiment was 9.97 min (range: 1.01–33.85 min, *SD* = 5.24). A total of 68 people completed within 5–20 min, accounting for 80%. Since participants were recruited from universities, their monthly discretionary income averaged ¥1,541.18 (range: ¥0–10000; *SD* = 1325.24).

**TABLE 1:** Means, SD, and correlations of all variables in monetary donation experiment.

	Mean	*SD*	1	2	3	4	5	6
(1) Color (0 = red and 1 = blue)	0.48	0.50						
(2) Behavior pattern	0.12	0.33	−0.317**					
(3) Monetary donation	50.72	33.62	0.228*	0.219*				
(4) Gender	0.45	0.50	0.063	–0.134	0.253*			
(5) Age	25.06	3.91	0.232*	0.064	0.274*	0.132		
(6) Discretionary money a month	1541.18	1325.24	0.114	0.089	0.307**	0.139	0.286**	
(7) Time spent	598.11	314.56	–0.129	–0.027	0.106	0.105	–0.137	−0.011

*p < 0.05, **p < 0.01.

The gender effects in our correlation analysis (Table 1) showed that women donated more money to the charity than men (*p* < 0.05), which was consistent with the results in a previous analysis showing that women were more altruistic than men (Rand et al., 2016; Branas-Garza et al., 2018). Age was also positively (*p* < 0.05) correlated with donations. One study on age-related bias in charitable giving reached the conclusion that older adults were more likely to donate to charity because they derived more positive emotions from it than younger people (Bjälkebring et al., 2016). The positive relationship (*p* < 0.01) between discretionary money and donations was also significant because of the correlation between age and discretionary money.

There was no significant correlation between the amount of time spent and the amount donated. We also compared the time spent in different colors; the average exposure time varied little (red: 10.41 min without one extremum = 200.86 min; blue: 9.57 min). Thus, the effects of the exposure time were similar under both conditions (red and blue).

**FIGURE 2:**
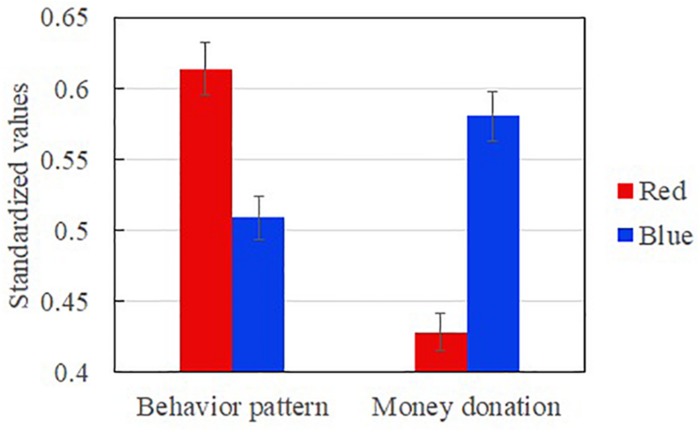
Behavioral patterns and monetary donations as a function of color.

To investigate the main effects of colors on donations, a one-factor, between-subjects ANOVA with the mediator of behavior pattern was conducted. An ANOVA with donation money as the dependent measure and behavioral pattern and color (red, blue) as the predictors revealed a significant mechanism. The ANOVA on donation money showed that participants donated significantly less in the red (M_red_ = 42.83, *n* = 41) than in the blue [M_blue_ = 58.14, *n* = 44, *F*(1,84) = 4.59, *p* = 0.03, Cohen’s *d* = 0.47; see Figure 2]. The ANOVA on behavioral patterns showed that participants were in a significantly more alternative-based process in the red (M_red_ = 0.22) than in the blue [M_blue_ = 0.01, *F*(1,84) = 9.26, *p* = 0.00, Cohen’s *d* = 0.67; see Figure 2]. No differential effects on the dependent measures were observed with gender, age, discretionary money, or time as covariates. The results showed that red caused a more alternative-based process than blue.

**FIGURE 3:**
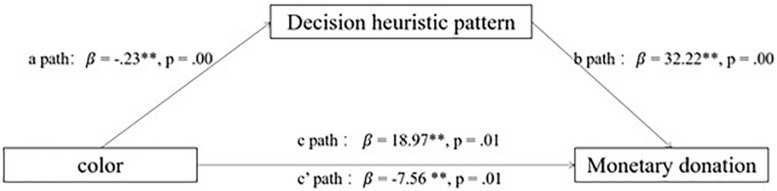
Mediation analysis.

The bootstrap method was then conducted in mediation analyses, with the decision heuristic pattern used as the mediator and four variables (gender, age, discretionary money, and time) used as covariates (Model 4; Hayes, 2013). The results showed that color affected the decision heuristic (a path: β = −0.23, *SE* = 0.07; *p* = 0.00; see Figure 3), and the pattern affected the degree of donation (b path: β = 32.22, *SE* = 10.45; *p* = 0.00; see Figure 3). A bootstrap analysis with 5,000 samples explained that the indirect effect of color on donation through pattern was negative and significant (c′ path: β = −7.56, *SE* = 3.16, 95%, CI −15.31, −2.62; see Figure 3). The direct effects of color on donation remained significant (c path: β = 18.97, *SE* = 7.03; *p* = 0.01; see Figure 3) when controlling for the mediator path. In addition, the results of Sobel tests for the indirect effect on the mechanism were significant (*z* = −2.20, *p* < 0.05).

In addition, a similar replicating experiment (*n* = 88) was conducted as a robustness test to determine whether the effect could be generalized to time donations. In the time donation experiment, the age of the participants ranged from 18 to 42 (mean = 25.56, *SD* = 4.17), and 48% (*n* = 42) of them were female. The average time to complete the experiment was 9.96 min (range: 1.26–24.82 min, *SD* = 4.65). A total of 71 people completed the test within 5–20 min, accounting for 81%. The tightness of their schedules averaged 4.59 on the Likert-7 scale (range: 2–7; *SD* = 1.44). The correlation between gender and time donation or age was not significant. Thus, when it came to donation time, there was no significant difference in the willingness of men and women, either old or young. In addition, schedule tightness was negatively correlated with time donations (*p* < 0.05). There was no significant correlation between the amount of time spent and the amount donated. We also compared the time spent in different colors; the average exposure time varies little (red: 10.07 min; blue: 9.85 min). Thus, the effects of the exposure time were similar under both conditions (red and blue).

An ANOVA on donation time showed that participants donated significantly less in the red than in the blue [M_red_ = 4.82, *n* = 45; M_blue_ = 6.74, *n* = 43; *F*(1,87) = 4.70, *p* = 0.03, Cohen’s *d* = 0.46]. An ANOVA on behavioral patterns showed that participants were in a significantly more alternative-based process in the red than in the blue [M_red_ = 0.17; M_blue_ = 0.00, *F*(1,87) = 7.95, *p* = 0.00, Cohen’s *d* = 0.61]. No differential effects on the dependent measures were observed with gender, age, schedule tightness, or time as covariates. The bootstrap method was then conducted in mediation analyses, with the decision heuristic pattern used as the mediator and four variables (gender, age, schedule tightness, time) used as covariates (Model 4; Hayes, 2013). The results showed that color affected the decision heuristic (a path: β = −0.19, *SE* = 0.06; *p* = 0.00), and this pattern affected the degree of donation (b path: β = 4.24, *SE* = 1.59; *p* = 0.01). A bootstrap analysis with 5,000 samples showed that the indirect effect of color on donation through pattern was negative and significant (c′ path: β = −0.82, *SE* = 0.44, 95%, CI −1.88, −0.15). The direct effects of color on donation remained significant (c path: β = 2.60, *SE* = 0.97; *p* = 0.01) when controlling for the mediator path. The results from the replicating experiment on time donation are consistent with the prior study. However, we noticed that the effect of time was weaker than money (see Supplementary Appendix), although people typically prefer to donate time over money to charities (Reed et al., 2007).

## Discussion

We investigated the influence of color (red vs. blue) on altruism. When shown the blue background, people showed more willingness to donate to the charity than when showed the red background. Through a gamble test, we found that red induced a more alternative-based process in information acquisition mode, which related to a more concrete logic compared with blue. This indicated that altruism is weakened in a more concrete mode of thinking, which is also consistent with previous conclusions (Yi et al., 2016). We showed these effects in two laboratory experiments (monetary donation and time donation). Money and time, from two donation dimensions, increased the universality and generality of our conclusion. We also found significant correlations between gender, age, and willingness to donate money, whereas no significant correlations were found between gender, age, and willingness to donate time.

We strived to make valuable research contributions with this study. First, we showed the different effects of red and blue on altruism. Specifically, blue boosted altruism compared to red. On the basis of previous literature on the influence of color on emotion (Mehta and Zhu, 2009), we added to prior work by focusing on altruistic behavior. One thing to note here is that in our experiment, the participants were only presented with one choice of donation charity. Although we chose a charity theme as universal as possible, is the single option was still a limitation of this study. In addition, no white or gray background was set as a control group. In future studies, a control group will be set to better understand the effects of red and blue.

Second, we identified behavioral pattern to be the mediating mechanism through which color affected altruism. Specifically, people with red backgrounds were more likely to access information in an alternative-based way, which our results show undermines altruism. In our study, we used a gamble test as the measure of behavior patterns. Two points worth noting here are that we did not collect data on whether participants had gambling habits or other potential gambling problems, which was another limitation of this study. In addition, the amount of gambling gains may have affected the subsequent willingness to donate, which was another limitation of this study. However, in the replication experiment (time donation), the influence of gambling money gain on subsequent donation time was small, and the results were still significant, which showed the generality of the conclusion.

Previous literatures of altruism mostly focus on the external factors, such as additional rewards (Ashraf et al., 2014) and making altruistic behaviors observed by others (Ekström, 2012). Our study expands the mechanisms through the behavioral patterns, thus advances the current research on the effects of color on cognition and behavior (e.g., Hatta et al., 2002; Mehta and Zhu, 2009; Bagchi and Cheema, 2013). More than that, these findings have more important practical applications for daily life. Color as a low-cost and easy to implement way can significantly affect human behavior patterns and thus affect altruism. Specifically, different colors may be beneficial for information acquisition of different tasks. For example, studying art (or another task that need a more attribute-based way to acquire information) might be better in a blue environment, while studying math (or another task that need a more alternative-based way to acquire information) in a red environment. Moreover, we can subtly promote altruism through information acquisition. Charitable products, for example, need to focus more on design than pricing. Given the importance of altruism for the well-being of our society, the mechanisms behind it are found to be conducive to the advancement of many major social issues in the future, such as environmental protection, resource recycling, social inequality and climate change.

Third, we adopted the two donation dimensions of money and time to further demonstrate the effect of red and blue on altruism. The difference between red and blue was greater when donating money. What’s more, we found that when it came to donating money, women were more altruistic, and older people were more compassionate and altruistic, findings that are consistent with previous studies (Bjälkebring et al., 2016; Rand et al., 2016; Branas-Garza et al., 2018). When it came to donating time, the effects of age and gender were no longer significant. The final limitation of our study was that there was no use of real money; the use of real money in further experiments will serve as a better indicator of altruistic behavior. This study demonstrated the consistency of color’s influence on both the time and money through two experiments. Further studies may include more altruistic behaviors to promote the conclusion drawn in this study.

Our results suggested that exposure to the background color of a web page affects behavioral patterns and altruistic tendencies. Changing background colors is easy, and charities can promote altruism by exposing potential donors to the color blue. Moreover, companies can influence consumers subtly by exposing them to different colors for their own purposes. For example, red may be more appropriate when companies want consumers to spend more on themselves.

In addition, the demand characteristics of different people may have had an impact on the results. Individual characteristics can affect physiological response; for example, a study showed that individual ability could significantly weaken the influence of background color (Dijkstra et al., 2008). So, more characteristics should be considered in future studies. In addition, participants in this study were all from China, so it is not clear whether the results would apply to people from different cultural backgrounds. The effects of color may be influenced by different cultures that have different experiences with certain colors (e.g., the association between colors and political parties in the United States). Moreover, a previous study posited that the influence of colors should be based on physiological rather than cultural factors (Gorn et al., 1997). In future studies, more control variables should be considered.

Color appears everywhere, and the effects of color still remain to be explored, especially on human behavior. Our study represented a small start, but has great implications for businesses, public welfare, and human practices.

## Data Availability Statement

The datasets generated for this study are available on request to the corresponding author.

## Ethics Statement

This study was approved by the human research ethics committee for non-clinical faculties at Nanjing University. The experiments in this study did not involve clinical trials, and all participants participated in the experiment with their safety, health, and rights protected. Moreover, all participants signed an informed consent form and volunteered to participate in the experiment; they could choose to quit at any time during the experiment. In the experiment, the computer screen served as a background color based on a color setting standard in previous research that has been found to pose no damage to the human body. All the participants had to do was click the mouse on a colored background to make a series of choices and predict their own behavior.

## Author Contributions

HL and JT developed the study concept. All authors contributed to the study design, data interpretation, and approved the final version of the manuscript for submission. XN collected the data and performed the data analysis with the help of JF and PW. XN drafted the manuscript under the supervision of HL, and JT provided the critical revisions.

## Conflict of Interest

The authors declare that the research was conducted in the absence of any commercial or financial relationships that could be construed as a potential conflict of interest.
