# Supplementary material for: Nudging Altruism by Color: Blue or Red?
Source: Front Psychol. 2020 Jan 22;10:3086. doi: 10.3389/fpsyg.2019.03086 (PMC6988519; doi:10.3389/fpsyg.2019.03086)

Appendix:

Instructions for the gambling test

Next comes the gamble test, where each set presents a matrix of four plans (plan A, B, C, D) to choose from, each plan consisting of four outcomes. The probabilities of the four outcomes appear in the first row, and the returns related to each outcome appear in the second row to the fifth row (corresponding to plan A, B, C, D). All plans have the same four outcome probabilities, and the final payoff for each plan is determined by the weighted sum of the outcomes according to the probabilities. Note that all values are "hidden" in the label boxes. When the cursor enters the box, the corresponding value appears until the cursor moves out. You can only read one box at a time. Finally, choose the plan that you think will benefit the most.

You will conduct a total of five sets of gamble test.

Red background in gamble test

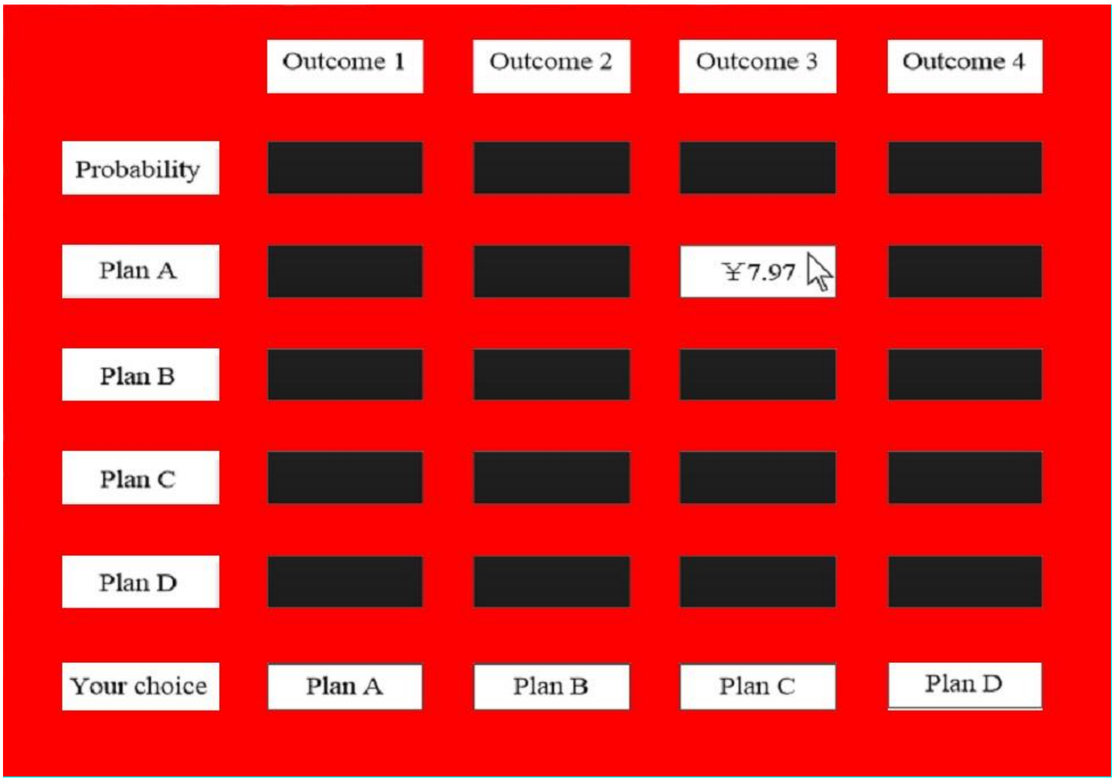

Blue background in gamble test

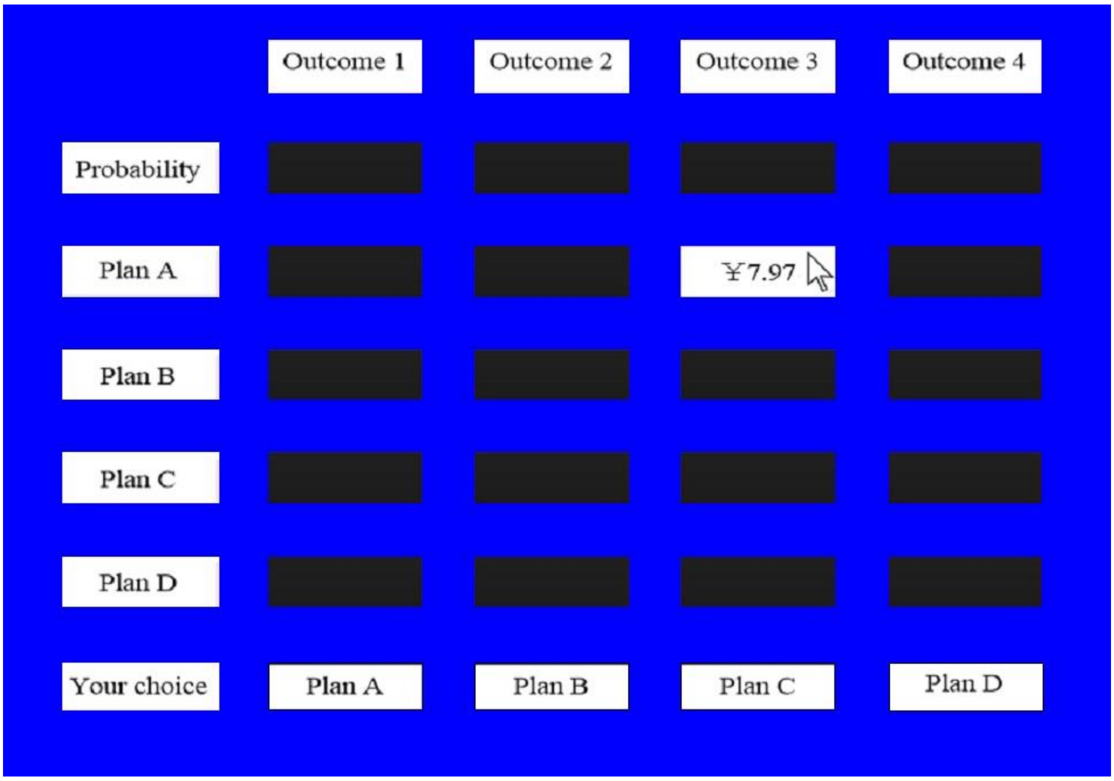

Red background in money donation questions

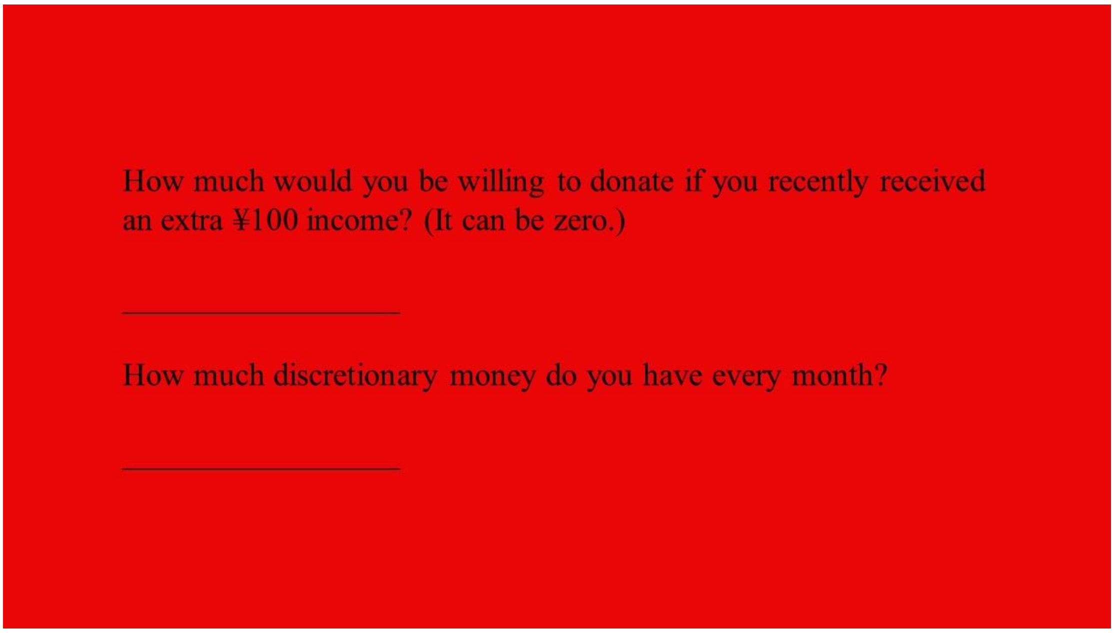

Blue background in money donation questions

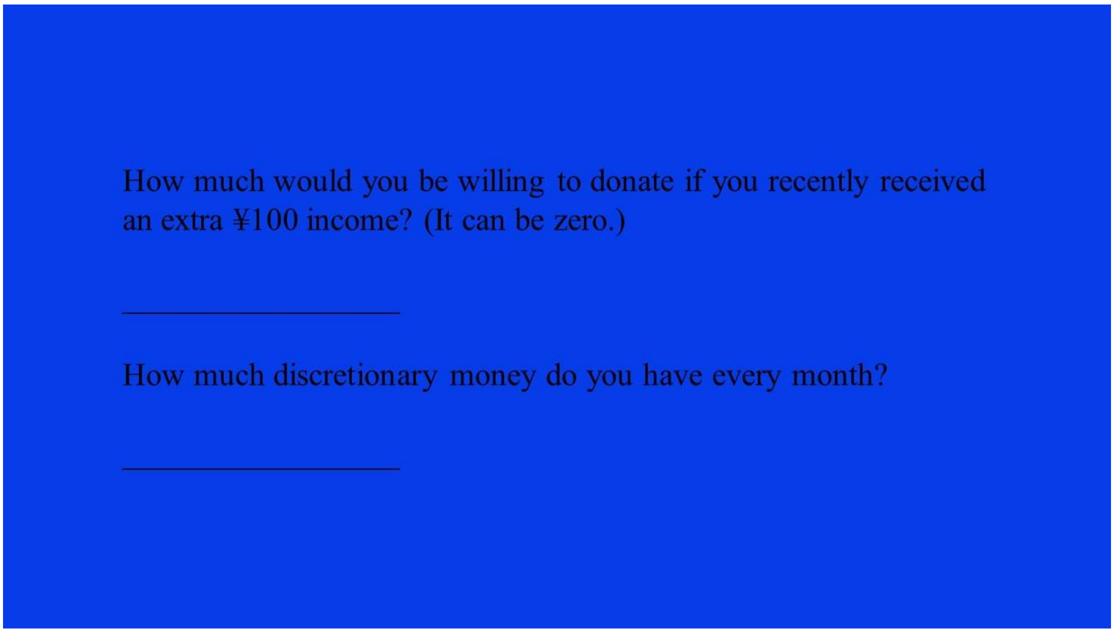

The picture before donation questions

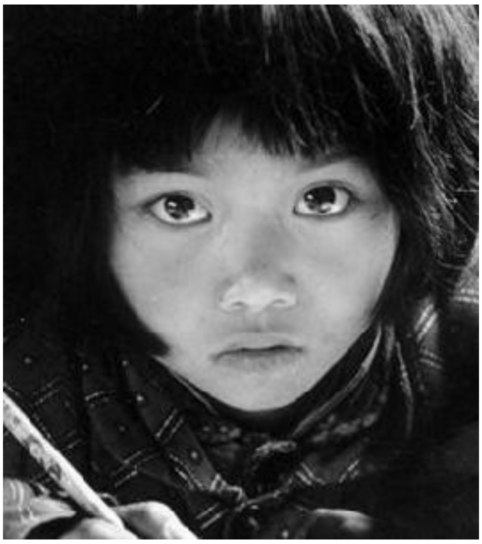

Means, SD, correlations of all variables in time donation experiment

	Mean	SD	1	2	3	4	5	6
1. color (0 = red and 1 = blue)	.51	.50						
2. behavior pattern	.09	.29	-.291**					
3. time donation	5.76	4.24	.228*	.198				
4. gender	.48	.50	-.069	-0.152	.119			
5. age	25.56	4.17	-.296**	.086	0.016	.119		
6. schedule tightness	4.59	1.44	-.277**	-.058	-.237*	.003	.019	
7. time spent	597.76	278.89	-0.024	.135	0.076	0.037	-0.152	0.091

*p<0.05, **p<0.01

Mediation analysis.in time donation experiment

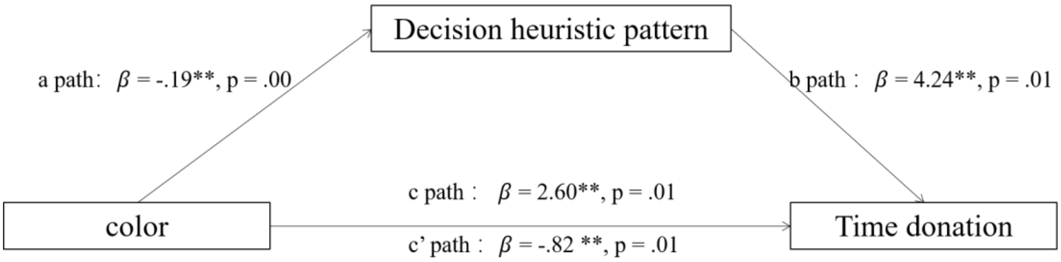

Behavior pattern and money donation as a function of color in time donation experiment

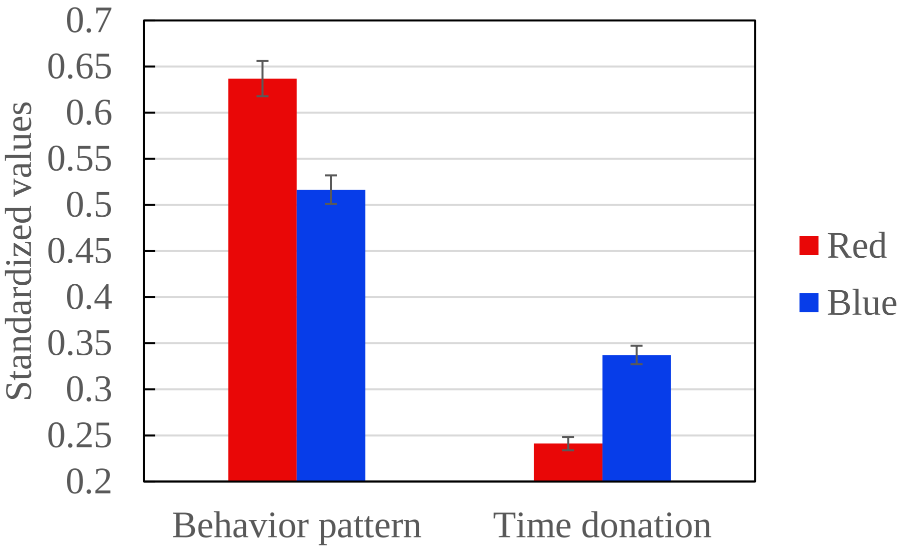

Two types of donation as a function of color.

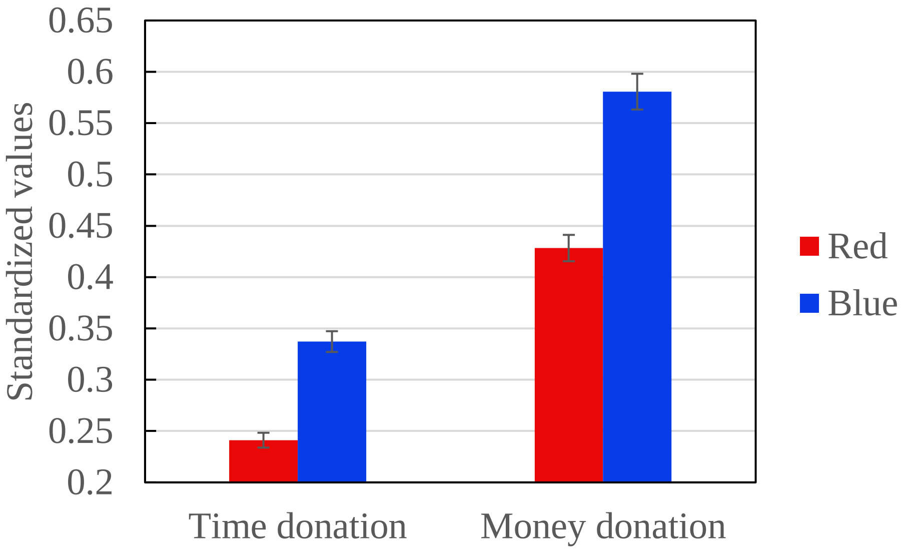

Supplement: Supplementary file 1 [file Data_Sheet_1.pdf]
